# Exposition de l’homme aux éléments traces métalliques et altération du sperme: étude menée dans les zones minières du Haut-Katanga en République Démocratique du Congo

**DOI:** 10.11604/pamj.2018.30.35.13694

**Published:** 2018-05-16

**Authors:** Richard-A-Mutshimbe Mukendi, Célestin Lubaba Nkulu Banza, Clarence-A-Kaut Mukeng, Jules Thaba Moyambe Ngwe, Albert Ntambwe-A-Nkoy Mwembo, Prosper Muenze Kayamba Kalenga

**Affiliations:** 1 Département de Gynécologie-obstétrique, Cliniques Universitaires de Lubumbashi, Faculté de Médecine, Université de Lubumbashi, République Démocratique du Congo; 2 Unité de Toxicologie et Environnement, Ecole de Santé Publique, Faculté de Médecine, Université de Lubumbashi, République Démocratique du Congo; 3 Unité de Biostatistique, Ecole de Santé Publique, Faculté de Médecine, Université de Lubumbashi, République Démocratique du Congo

**Keywords:** Eléments traces métalliques, baisse de la fertilité, zones minières, Metal trace elements, fertility decline, mining areas

## Abstract

**Introduction:**

L'exposition à l'arsenic et au cadmium entraine des effets néfastes importants. L'objectif de cette étude est de déterminer les concentrations urinaires en arsenic et en cadmium chez les hommes et d'analyser leur profil en rapport avec les éléments du spermogramme.

**Méthodes:**

Il s'agit d'une étude analytique exposés-non exposés où les hommes exposés ont été recrutés dans une zone minière du Haut-Katanga, en RDC et les hommes non-exposés dans une zone loin de toute activité minière.

**Résultats:**

Nos résultats montrent que 48% des sujets exposés ont un pH du sperme en dessous de la valeur seuil de 7,2 contre seulement 16% des sujets non-exposés. Le risque d'abaissement du pH en deçà des normes étant de plus de 4 fois supérieur (OR=4,85 [1,9-12,39]) chez les exposés. La différence est statistiquement significative entre les moyennes du nombre global des spermatozoides à l'avantage des sujets non-exposés et celles des spermatozoides anormaux beaucoup plus chez les sujets exposés. On note une dégradation plus rapide de la mobilité des spermatozoides chez les sujets exposés. Par ailleurs, 44% des hommes exposés ont une concentration urinaire en arsenic > 20 µg/l contre 8% des sujets non-exposés, le risque d'accumulation excessive d'arsenic est neuf fois plus élevé chez les hommes exposés que chez ceux non exposées (OR=9,04 [2,82-28,96]). Soixante pourcents des hommes exposés ont une concentration urinaire en cadmium ≥ 0,5 µg/ml contre 38% des sujets non-exposés avec un Odd Ratio de 2,45 [1,1-5,47], traduisant un risque d'accumulation excessive de cadmium chez les hommes exposés. D'autre part, on constate chez les hommes exposés que les fortes concentrations urinaires en arsenic et en cadmium entrainent une dégradation proportionnelle des éléments du spermogramme.

**Conclusion:**

Le présent travail montre bien d'une part de fortes concentrations urinaires d'arsenic et de cadmium et d'autre part l'altération plus rapide et plus sévère des éléments du spermogramme chez les hommes vivant en zone minière, suggérant une baisse de la fertilité masculine qui mérite d'être davantage documentée dans les travaux futurs.

## Abstract (en)

**Introduction:**

Arsenic and cadmium exposures cause significant adverse effects. This study aims to determine the urinary arsenic and cadmium concentrations in men in order to analyze their profile in relation to sperm count test values.

**Methodes:**

We conducted an exposed-non-exposed analytical study of men exposed to arsenic and cadmium in a mining area in Upper Katanga, DRC and of non-exposed men living far from any mining area.

**Results:**

Study results show that 48% of exposed subjects had semen pH below the threshold value of 7.2 versus 16% of non-exposed subjects. The risk of a lowering of pH below the standards was more than 4 times higher (OR=4.85[1.9-12.39]) among exposed men. The difference between the averages of the total number of spermatozoa was statistically significant to the advantage of non-exposed subjects. Abnormal number of spermatozoa was much higher in exposed subjects. It was recorded a more rapid spermatozoa mobility degradation in exposed subjects. In addition, 44% of exposed men had urinary arsenic concentration > 20 µg/L versus 8% of non-exposed subjects, the risk of excessive arsenic accumulation was nine times higher in exposed men than in non-exposed men (OR=9.04 [2.82-28.96]). Sixty percent of exposed men had urinary cadmium concentration ≥0.5µg/ml versus 38% of non-exposed subjects, with an Odd Ratio of 2.45 [1.1-5.47], reflecting a risk of excessive cadmium accumulation in exposed men. On the other hand, it was recorded that, among exposed men, high urinary arsenic and cadmium concentrations resulted in a proportional degradation of sperm count test values.

**Conclusion:**

This study shows, on one hand, high urinary arsenic and cadmium concentrations and, on the other hand, more rapid and more severe alterations of sperm count test values in men living in mining area. This suggests a decline in male fertility which deserves to be further documented in future studies.

## Introduction

La fertilité est la capacité biologique à se reproduire. Elle se distingue de la fécondité, celle-ci étant une notion démographique faisant référence à la présence ou absence de conceptions abouties et fortement influencée par la contraception. Chez l'homme, la fertilité peut être estimée par l'examen quantitatif et qualitatif du sperme ainsi que par l'évaluation du bilan sanguin des hormones de la reproduction [1]. Il est admis actuellement que des facteurs toxicologiques de l'environnement sont à la base de la dégradation de la fertilité tant masculine que féminine [2-4]. Lambrot et al. [5] évoquent l'idée selon laquelle les polluants environnementaux auraient un effet perturbateur du système endocrinien pendant «les fenêtres critiques du développement» c'est-à-dire chez les femmes enceintes, les nourrissons, les jeunes enfants et les adolescents pendant la puberté. Durant ces périodes, de très faibles perturbations du système endocrinien peuvent dérégler la mise en place des organes reproducteurs et perturber la fonction reproductrice, cela de générations en générations. Alvarez [6] rappelle que trois phénomènes majeurs récurrents ces dernières années ont attiré l'attention des épidémiologistes. Il s'agit de l'augmentation des malformations uro-génitales masculines à la naissance dont l'hypospadias et la cryptorchidie, l'augmentation du cancer des testicules ainsi que la diminution de la qualité du sperme. Dans une étude parue en 2007, Parenteau [7] soutient que les substances reprotoxiques contaminent la chaine alimentaire, par le mécanisme de bioaccumulation, et augmentent en concentration d'un maillon trophique à l'autre par le phénomène de bioamplification. L'homme, en tant que super prédateur situé au sommet de la pyramide écologique, présente souvent les concentrations les plus élevées avec des effets délétères notamment sur la fonction de reproduction.

En 2011, Bied Damon et Mirakian [8] avançaient la thèse selon laquelle les principaux polluants chimiques toxiques pour la fertilité agissent par effet perturbateur endocrinien et mutagène alors que les solvants organiques le font par un effet génotoxique. Les effets délétères des polluants se feraient ressentir à différents niveaux de la reproduction: la spermatogénèse, le développement ovocytaire, le contrôle hormonal de la fonction de reproduction, la fécondation, l'implantation embryonnaire et le fœtus. La plupart des toxicologues et médecins œuvrant dans le secteur de la santé de la reproduction s'accordent sur le fait que parmi les polluants reprotoxiques, les éléments traces métalliques (ETM) jouent un rôle majeur. Bien que les recherches ne soient pas concluantes sur les effets délétères de l'ensemble des ETM sur les organes génitaux et la capacité de reproduction, cependant l'unanimité tend à se réaliser sur les effets reprotoxiques de quatre d'entre-eux: l'arsenic, le mercure, le cadmium et le plomb [2, 9-13]. De nombreuses études menées au Katanga ont démontré des concentrations anormalement élevées de la plupart des ETM dans l'eau, le sol, l'air, les cheveux et liquides biologiques humains comme le sang et les urines mais aussi dans diverses espèces aquatiques comme les poissons, denrées fort appréciées des populations vivant dans le Grand Katanga [14-17]. Dans une étude menée par Katemo et al en 20^10^ [14], huit ETM (Cu, Co, Zn, Cd, Pb, U, V et As) ont été retrouvés dans des échantillons d'eau, de plancton, de feuilles de Phragmites australis ainsi que de muscles et de branchies de trois espèces des poissons du bassin de la Lufira supérieure, aux environ de la ville de Likasi dans le Haut-Katanga, en RDC. Les résultats indiquent de fortes concentrations en cuivre et cobalt dans les effluents du complexe hydrométallurgique de Shituru. Si la contamination des cours d'eau diminue avec l'éloignement de la source de pollution, les valeurs sont très élevées dans le lac Tshangalele pour le plancton et les feuilles de P. australis. Pour les poissons, les résultats indiquent que le Pb, U, V, Cu, Co et Cd s'accumulent préférentiellement dans les branchies alors que le Zn s'accumule plus dans les muscles. L'As s'accumule dans le même ordre de grandeur dans les deux organes. Ces résultats confirment la pollution du bassin de la Lufira, aux environs de la ville de Likasi en RDC, par les effluents du complexe hydrométallurgique de Shituru à Likasi. Ainsi qu'en témoignent de nombreuses études [1, 8-12], les ETM dont l'arsenic et le cadmium retrouvés en fortes concentrations dans l'eau, les aliments (poissons et végétaux) et la matière biologique humaine au sud-est du Grand-Katanga ont des effets néfastes sur la fertilité chez les hommes. Cependant, ces effets ne sont pas documentés chez les habitants des zones à forte pollution minière et métallurgiques du Haut-Katanga, en RDC.

Ces différentes études se sont contentées d'établir un tableau descriptif de la situation rencontrée dans certaines contrées concernées par l'exploitation minière sans pour autant pousser les investigations jusqu'à effectuer une analyse comparative entre les zones exposées aux déchets miniers reprotoxiques et les zones exemptées du Grand Katanga. Nous pensons qu'une étude visant à déterminer les liens d'association entre les concentrations urinaires d'arsenic ainsi que du cadmium et l'altération du sperme des hommes vivant dans les zones minières du sud-est du Grand-Katanga serait opportune à l'heure de la mise en œuvre des objectifs du millénaire pour le développement durable. La présente étude a pour objectif de déterminer la qualité du sperme et des spermatozoïdes en fonction des concentrations urinaires en arsenic et en cadmium chez les hommes vivant dans les zones minières de la province du Haut-Katanga, en RDC.

## Méthodes

Il s'agit d'une étude transversale à visée analytique: exposés et non-exposés. Les exposés ont été recrutés dans des villages riverains du bassin de la Lufira supérieure où les eaux de lavage des minerais et les effluents des usines de la ville de Likasi contenant des ETM sont rejetés sans traitement préalable tandis que les non-exposés ont été recrutés dans une zone agricole éloignée de toute activité minière à savoir les environs de la ville de Kalemie à raison d'un non-exposé pour un exposé. Les sujets de l'étude sont recrutés dans la tranche d'âge allant de 18 à 40 ans. L'échantillonnage a été aléatoire de convenance tant pour les exposés que pour les non-exposés. Ont été inclus dans l'étude les hommes habitant le site concerné depuis au moins 10 ans et ayant marqué leur accord pour participer à l'étude. Ont été exclus de l'étude les hommes présentant une étiologie évidente de stérilité ou actuellement sous traitement contre la stérilité ou de toute autre forme de procréation médicalement assistée. Pour les études multivariées, on recommande au moins 5 sujets par variable explicative considérée, soit pour notre étude un minimum de 50 hommes dans le groupe des exposés et 50 autres hommes dans le groupe des non-exposés soit en tout 100 hommes. Les paramètres d'étude sont les concentrations urinaires en arsenic et en cadmium, le pH du sperme, la numération globale des spermatozoïdes par champs, la numération des spermatozoïdes anormaux par champs, la numération des spermatozoïdes mobiles par champs après une heure et après six heures du prélèvement. L'analyse de nos résultats s'est faite à l'aide des statistiques usuelles, des Odds Ratio avec intervalles de confiance au seuil de 95% et des tests d'association calculés grâce au logiciel SPSS version 17. Les échantillons ont tous été analysés dans le Laboratoire de Toxicologie industrielle et l'Unité de Médecine du travail (Université catholique de Louvain, Belgique) en analyse aveugle. Dans tout échantillon d'urine, les ETM dont l'arsenic et le cadmium étaient quantifiés au moyen de la méthode ICP-MS avec un appareil de marque *Agilent 7500 ce*. Les résultats de nos recherches sont présentés sous forme de tableaux et de figures. La présente étude a été approuvée par le comité d'éthique médical de l'Université de Lubumbashi en Novembre 2014.

## Résultats

**Caractéristiques du spermogramme selon les sites pH du sperme:** Le Tableau 1 montre que 48% des sujets exposés ont un Ph du sperme en dessous de la valeur seuil qui est de 7,2 tandis que seulement 16% des sujets non-exposés sont retrouvés dans la tranche en deçà des normes. L'Odd Ratio calculé au seuil de 95% est de 4,85 [1,9-12,39], montrant que le fait de vivre dans un site pollué par l'exploitation minière est associé à un risque de diminution du Ph du sperme en deçà des valeurs normales de près de 5 fois supérieur comparativement au fait de vivre dans un site éloigné de toute activité minière.

**Tableau 1:** Distribution des sujets selon le Ph du sperme

**pH du sperme Sujets**	< 7,2	≥ 7,2	OR (IC 95%)
**Exposés**	24 (48%)	26 (52%)	4,85 (1,9 – 12,39)
**Non-exposés**	8 (16%)	42 (84%)	1,00
**Total**	32(32%)	68 (68%)	-

**Numération des spermatozoïdes par champ:** L'analyse du Tableau 2 révèle que 94% d'hommes exposés ont entre 11 et 19 spermatozoïdes par champ tandis que seulement 78% d'hommes non exposés ont un nombre des spermatozoïdes par champ dans cette fourchette. La moyenne du nombre des spermatozoïdes par champ chez les non-exposés est de 15,64±4,019 tandis que celui des exposés est de 13,08±2,059. Le test T de Student montre une différence statistiquement significative entre les deux moyennes à l'avantage des non-exposés. Le constat est donc qu'il existe une nette tendance à la baisse du nombre global des spermatozoïdes dans les sites concernés par la pollution minière. En rapport avec le nombre des spermatozoïdes anormaux par champ nous avons recensé 64% d'hommes non exposés contre 78% d'hommes exposés (Tableau 2). Le nombre moyen des spermatozoïdes anormaux par champ chez les non-exposés est de 7,40±2,060 tandis que celui des exposés est de 8,68±1,168. Le test T de Student montre une différence statistiquement significative entre les deux moyennes avec une prépondérance chez les exposés. On observe ainsi une nette tendance à la hausse du nombre des spermatozoïdes anormaux dans les zones impliquées dans des exploitations minières. Le Tableau 2 indique que chez 54% d'hommes non exposés plus de 10 spermatozoïdes sont encore mobiles une heure après le prélèvement tandis que seulement 4% d'hommes exposés avaient encore plus de 10 spermatozoïdes mobiles par champ après une heure du prélèvement. Le nombre moyen des spermatozoïdes mobiles par champ dans l'heure qui suit le prélèvement chez les non-exposés est de 9,50±2,525 tandis que celui des exposés est de 5,56±1,981. Le test T de Student montre une différence statistiquement significative entre les deux moyennes en faveur des non-exposés. On assiste donc à une baisse importante de la mobilité des spermatozoïdes endéans l'heure qui suit le prélèvement chez les sujets vivant dans les zones minières. Six heures après le prélèvement chez 68% d'hommes non exposés on répertoriait entre 1 et 3 spermatozoïdes encore mobiles contre seulement chez 40% d'hommes exposés (Tableau 2). Le nombre moyen des spermatozoïdes mobiles par champ au-delà de 6h après le prélèvement chez les non-exposés est de 1,72±1,325 tandis que celui des exposés est de 0,60±0,833. Le test T de Student montre une différence statistiquement significative entre les deux moyennes à l'avantage des non-exposés. Il est donc évident que la durée de vie des spermatozoïdes est fortement altérée chez les sujets vivant dans les espaces envahis par l'exploitation minière.

**Tableau 2:** Distribution des sujets selon le nombre des spermatozoïdes

Nombre des spermatozoïdes par champs	Exposés (n=50)	Non-exposés (n=50)	Total (n=100)
**Global**			
≤ 10	3 (6%)	5 (10%)	8 (8%)
11 à 19	47 (94%)	39 (78%)	86 (86%)
≥ 20	0 (0%)	6 (12%)	6 (6%)
**Moyenne (ET)**	**13,08±2,06**	**15,64±4,02**	**T de student : p< 0,05**
Anormaux			
≤ 5	0 (0%)	8 (16%)	8 (8%)
6 à 9	39 (78%)	32 (64%)	71 (71%)
≥ 10	11 (22%)	10 (20%)	21 (21%)
**Moyenne (ET)**	**8,68±1,17**	**7,40±2,06**	**T de student : p< 0,05**
Mobiles après 1h			
≤ 5	26 (52%)	4 (8%)	30 (30%)
6 à 9	22 (44%)	19 (38%)	41 (41%)
≥ 10	2 (4%)	27 (54%)	29 (29%)
**Moyenne (ET)**	**5,56±1,98**	**9,50±2,52**	**T de student : p< 0,05**
Mobiles après 6h			
0	28 (56%)	12 (24%)	40 (40%)
1 à 3	20 (40%)	34 (68%)	54 (54%)
≥ 4	2 (4%)	4 (8%)	6 (6%)
**Moyenne (ET)**	**0,60±0,83**	**1,72±1,32**	**T de student : p< 0,05**

**Détermination des concentrations urinaires d'arsenic et de cadmium dans la population masculine étudiée Concentrations urinaires en arsenic:** Le Tableau 3 montre que 44% des sujets exposés ont une concentration urinaire en arsenic au-delà de la valeur seuil qui est de 20 µg/l contre seulement 8% des sujets non-exposés qui se retrouvent avec des concentrations urinaires en arsenic au dessus de 20 µg/l. L'Odd Ratio calculé au seuil de 95% est de 9,04[2,82-28,96], montrant que vivre dans un site pollué par l'exploitation minière est associé à un risque de dépassement des concentrations urinaires en arsenic au-delà des valeurs normales de plus de 9 fois supérieur comparativement à ceux qui vivent dans un site éloigné de toute exposition aux ETM.

**Tableau 3:** Distribution des concentrations urinaires en arsenic en fonction du site

	Concentration urinaire en As		
Sujets	> 20 µg/l	≤ 20 µg/l	OR (IC 95%)
Exposés	22 (44%)	28 (56%)	9,04 (2,82-28,96)
Non-exposés	4 (8%)	46 (92%)	1,00
Total	26 (26%)	74 (74%)	-

**Concentrations urinaires en cadmium:** L'observation du Tableau 4 révèle que 60% des sujets exposés ont une concentration urinaire en cadmium égale ou supérieure à 0,5 µg/ml qui est la valeur seuil contre seulement 38% des sujets non-exposés. L'Odd Ratio calculé au seuil de 95% est de 2,45 [1,1-5,47]. La conclusion qui en découle est que le fait de vivre dans un site subissant la pollution due à l'exploitation minière est associé à un risque de dépassement des concentrations urinaires en cadmium au-delà des valeurs normales de plus de 2 fois supérieur en comparaison aux habitants des sites éloignés de toute activité minière.

**Tableau 4:** Distribution des concentrations urinaires en cadmium en fonction du site

	Concentration urinaire en Cd		
**Sujets**	**> 0,5 µg/ml**	**≤ 0,5 µg/ml**	**OR (IC 95%)**
**Exposés**	30 (60%)	20 (40%)	**2,45 (1,1-5,47)**
**Non-exposés**	19 (38%)	31 (62%)	**1,00**
**Total**	**49 (45%)**	**51 (51%)**	**-**

**Figure 1:**
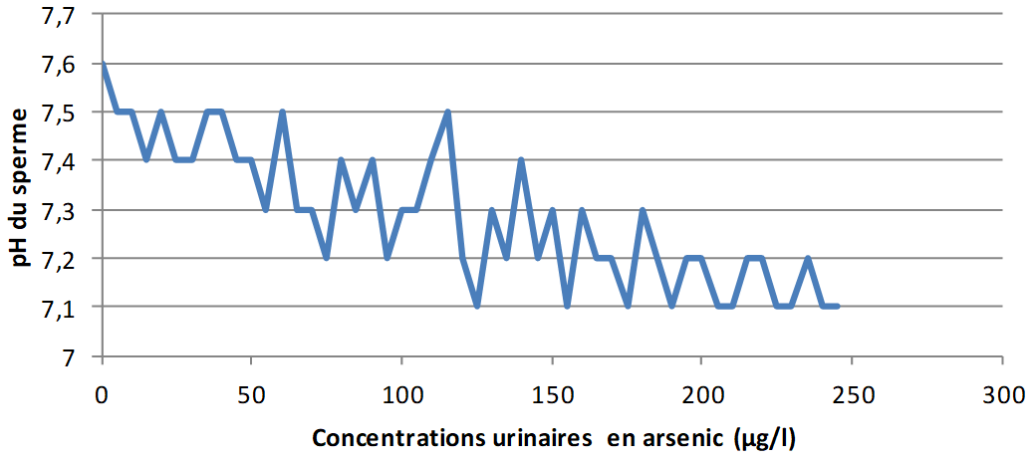
Concentrations urinaires en arsenic en µg/l versus Ph du sperme chez les sujets exposés

**Figure 2:**
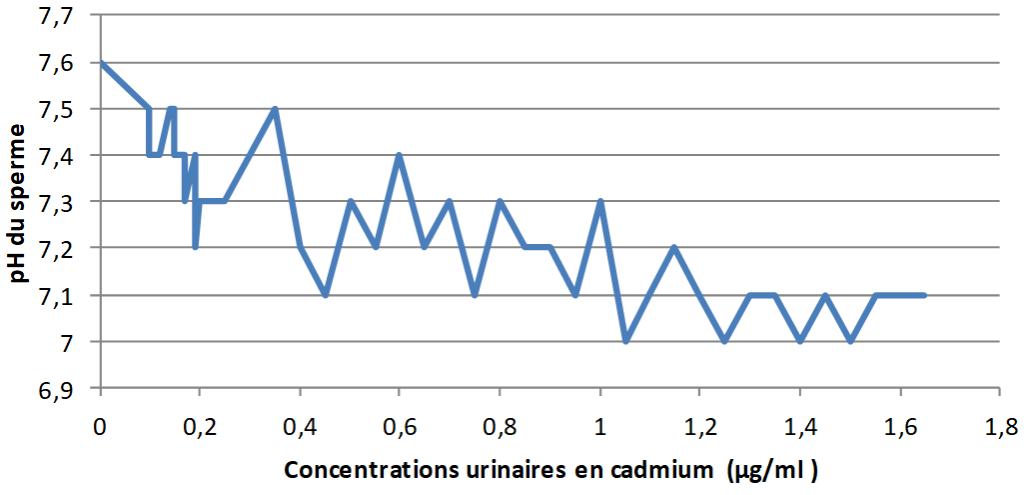
Concentrations urinaires en cadmium en µg/ml versus Ph du sperme chez les sujets exposés

**Figure 3:**
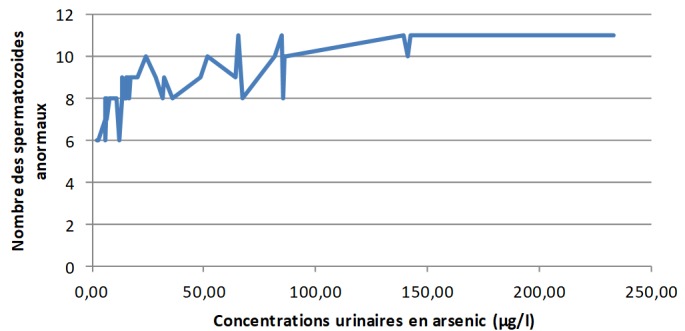
Concentrations urinaires en arsenic en µg/l versus nombre des spermatozoïdes anormaux par champs chez les sujets exposés

**Figure 4:**
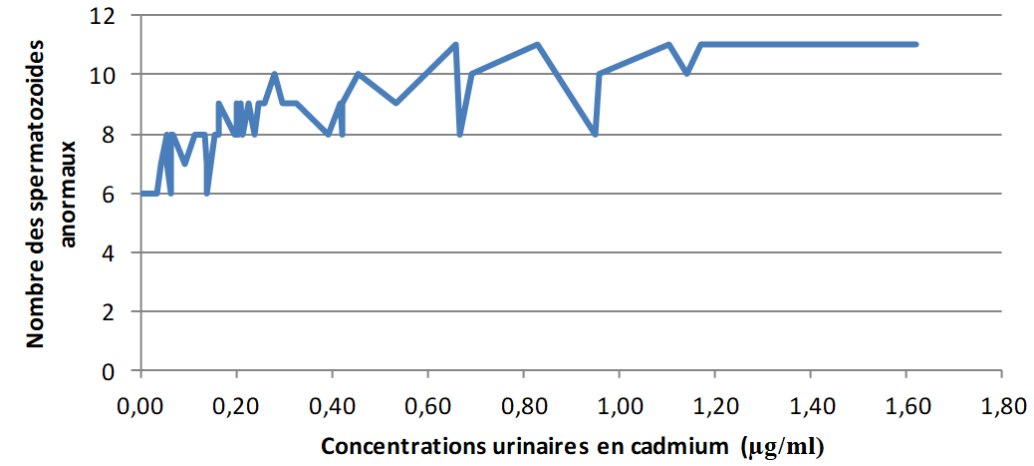
Concentrations urinaires en cadmium en µg/ml versus nombre des spermatozoides anormaux chez les sujets exposés

**Figure 5:**
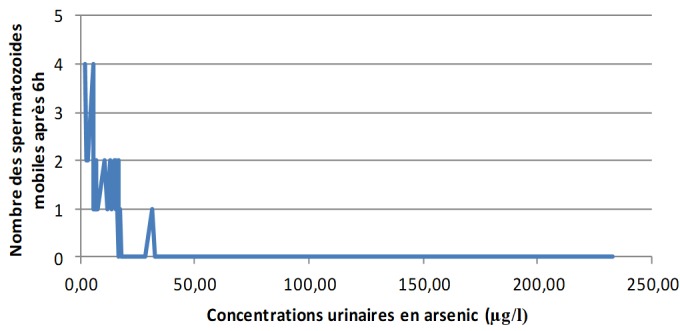
Concentrations urinaires en arsenic en µg/l versus nombre des spermatozoides mobiles après 6h chez les sujets exposés

**Figure 6:**
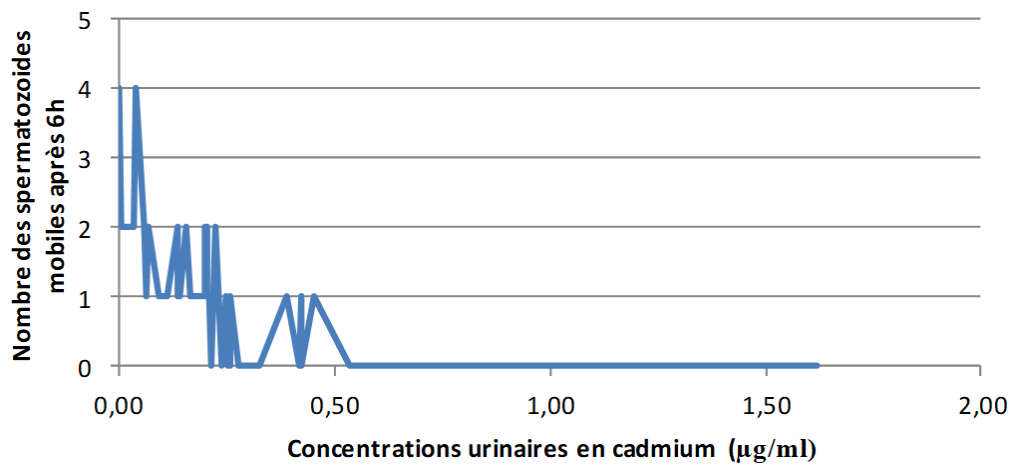
Concentrations urinaires en cadmium en µg/ml versus nombre des spermatozoides mobiles après 6h chez les sujets exposés

**Les concentrations urinaires en arsenic et en cadmium versus les éléments du spermogramme chez les sujets exposés:** L'analyse de la Figure 1 montre clairement qu'à mesure que les concentrations urinaires en arsenic s'élèvent, le pH du sperme perd de plus en plus de son alcalinité dans les zones minières du Haut-Katanga. La Figure 2 révèle que le pH du sperme diminue au fur et à mesure que les concentrations urinaires en cadmium augmentent dans les zones minières du Haut-Katanga. L'analyse de la Figure 3 révèle que le nombre des spermatozoïdes anormaux par champ augmente proportionnellement à l'élévation des concentrations urinaires en arsenic chez les hommes exposés. La Figure 4 montre qu'avec l'élévation des concentrations urinaires en cadmium le nombre de spermatozoïdes anormaux par champ augmente de plus en plus chez les hommes exposés. La Figure 5 renseigne que les fortes concentrations urinaires en arsenic ont tendance à entrainer une diminution plus rapide de la mobilité des spermatozoïdes chez les hommes exposés six heures après le prélèvement. La Figure 6 révèle que les fortes concentrations urinaires en cadmium tendent à déclencher une diminution rapide de la mobilité des spermatozoïdes chez les hommes exposés six heures après le prélèvement.

## Discussion

L'analyse des résultats de la présente étude met en évidence l'altération du sperme des hommes vivant dans le bassin de la Lufira supérieure aux environs de la ville de Likasi dans la province du Haut-Katanga, en RDC où les entreprises exploitant les minerais cobaltifères et cupriques rejettent sans traitement préalable les déchets dans l'environnement. Plusieurs études [14-17] ont révélé la présence des ETM en fortes concentrations au niveau tant de la faune que de la flore de cette partie du Grand-Katanga. Ces différentes études ont indiqué que parmi ces ETM on retrouve de façon constante le cadmium et l'arsenic, éléments répertoriés comme ayant un fort pouvoir reprotoxique. Des études récentes concernant l'exposition à l'arsenic et au cadmium ont confirmé la dégradation des éléments du spermogramme par les ETM reprotoxiques. En effet, ils agiraient à plusieurs niveaux pour perturber la fonction reproductive de l'homme. Notamment au niveau des cellules de Leydig en perturbant la sécrétion des androgènes par une peroxydation lipidique. Ils agissent également sur les cellules de Sertoli en altérant la spermatogénèse entrainant ainsi une oligozoospermie. Ils ont un effet délétère sur la morphologie et la stabilité de la chromatine des spermatozoïdes et entrainent des dommages graves au niveau de l'ADN des spermatozoïdes. La présence de ces éléments sous leur forme inorganique dans le sperme déclenche au niveau des vésicules séminales et de la prostate une altération de la vitalité et de la mobilité des spermatozoïdes [1]. Aux Etats-Unis d'Amérique, dans une vaste étude de cohorte menée sur 501 couples dans 4 comtés de l'Etat du Michigan et 12 comtés de l'Etat du Texas, les chercheurs ont abouti aux résultats très éloquents. Cette étude a démontré que la concentration urinaire de plomb, de cadmium et d'arsenic sont inversement proportionnelle au taux de chances de fécondabilité chez les hommes [10]. D'autres études ont montré que le cadmium pouvait entraîner une altération de la qualité des spermatozoïdes: altération de la réaction acrosomique et anomalie de la formation du cytosquelette. Une exposition excessive en cadmium a aussi été impliquée dans l'infertilité associée à la varicocèle [11].

L'exposition très importante à l'arsenic inorganique peut provoquer une stérilité chez l'homme. En effet, du fait de sa forte affinité avec le groupement thiol «-SH», l'arsenic inorganique séquestre ce groupement présent dans certains acides aminés qui constituent les protéines de la membrane du spermatozoïde et des microtubules axonémaux chez l'homme. La cible la plus importante est le groupement thiol de la tubuline, principal composant de l'axonème. L'autre mécanisme possible de toxicité est la génération de radicaux libres (ROS). L'exposition des spermatozoïdes à l'arsenic entraine également la diminution rapide des paramètres du mouvement [13]. Dans notre série, la diminution du nombre des spermatozoïdes mobiles dans l'heure qui suit le prélèvement s'accentue de façon remarquable au-delà de la sixième heure ce qui suggère la baisse de la longévité des spermatozoïdes pouvant s'expliquer par la présence de l'arsenic dans l'environnement pollué des zones minières du Haut-Katanga. La forte prévalence des spermatozoïdes anormaux dans les échantillons de sperme provenant des zones exposées pourrait présenter un lien avec la survenue de plus en plus fréquente des nouveau-nés présentant des malformations congénitales. En effet, Lubala et al [18] ont relevé une augmentation significative de la prévalence des malformations congénitales dans la ville de Lubumbashi et ses environs par rapports aux études antérieures; soit 5,84 pour 1000 naissances vivantes. Il a émis l'hypothèse selon laquelle l'intensification des activités minières et la pollution environnementale provenant des carrières, mines et usines de traitements des minerais seraient associée à la hausse de ce phénomène morbide. Pourtant dans la plupart des pays ayant des entreprises minières l'état prend toujours soin du fait que les différentes législations minières puissent prévoir dans leurs dispositions la prise en compte de l'aspect environnemental dans l'exécution des projets industriels. Malheureusement force est de constater que les services géologiques de la RDC, comme du reste dans la plupart des pays sous-équipés, ne disposent ni de moyens ni de spécialistes capables d'appréhender tous les aspects liés à l'étude et à la protection de l'environnement minier. Face à la matérialisation et à l'intensification de ces problèmes, il devient urgent d'intégrer désormais les exigences de la protection de l'environnement dans la politique de relance du secteur minier en RDC. Il s'agira de concilier la nécessité d'une production minière, génératrice de revenus et d'emplois pour l'économie nationale, et le désir légitime de maintenir un environnement sain dans nos pays car en négligeant cet aspect important l'avenir de plusieurs générations est hypothéqué par l'altération des organes reproducteurs de façon irréversible.

## Conclusion

A la lumière des résultats de nos investigations il apparait de façon claire que l'exploitation minière dans le Haut-Katanga au mépris de loi en la matière présentent des risques certains pour la santé reproductive des populations exposées à cette pollution environnementale. Le fait de vivre dans un site subissant la pollution due à l'exploitation minière est associé à un risque de dépassement des concentrations urinaires d'arsenic et de cadmium au-delà des valeurs normales respectivement de plus de 9 et 2 fois supérieurs en comparaison aux habitants des sites éloignés de toute activité minière. Les paramètres de la fertilité masculine sont fortement altérés chez les personnes victimes de cette pollution. Ainsi le pH perd de plus en plus son alcalinité à mesure que les concentrations urinaires d'arsenic et de cadmium s'élèvent. Le même constat est fait pour les autres paramètres du spermogramme en l'occurrence le nombre des spermatozoïdes et leur mobilité qui sont fortement altérés proportionnellement aux concentrations urinaires d'arsenic et de cadmium chez les hommes vivant dans les zones minières du Haut-Katanga. A l'heure de la mise en application des objectifs du millénaire sur le développement durable, une prise de conscience collective doit être de mise en vue d'enrayer la dérive vers une intensification des activités minières sans l'assurance de l'installation simultanée des usines connexes destinées à traiter les déchets industriels avant leur déversement dans les cours d'eau et les sols où ils contaminent les nappes phréatiques libérant ainsi dans l'environnement le cadmium et l'arsenic dont les effets reprotoxiques ne sont pas négligeables.

### Etat des connaissances actuelles sur le sujet

- Il est admis actuellement que des facteurs toxicologiques de l'environnement sont à la base de la dégradation de la fertilité tant masculine que féminine;
- L'arsenic et le cadmium agiraient à plusieurs niveaux pour perturber la fonction reproductive de l'homme, notamment au niveau des cellules de Leydig en perturbant la sécrétion des androgènes par une peroxydation lipidique;
- De nombreuses études menées au Katanga ont démontré des concentrations anormalement élevées de la plupart des éléments traces métalliques dans l'eau, le sol, l'air, les cheveux et liquides biologiques humains comme le sang et les urines mais aussi dans diverses espèces aquatiques comme les poissons.

### Contribution de notre étude à la connaissance

- Le fait de vivre dans un site subissant la pollution due à l'exploitation minière au Katanga est associé à un risque de dépassement des concentrations urinaires d'arsenic et de cadmium au-delà des valeurs normales en comparaison aux habitants des sites éloignés de toute activité minière;
- Les hommes victimes de la pollution minière ont le pH du sperme qui perd de plus en plus son alcalinité à mesure que les concentrations urinaires d'arsenic et de cadmium s'élèvent, altérant ainsi leur fertilité;
- Le nombre global des spermatozoïdes, leur mobilité et leur longévité sont fortement altérés proportionnellement aux concentrations urinaires d'arsenic et de cadmium chez les hommes vivant dans les zones minières du Katanga tandis que la proportion des spermatozoïdes anormaux augmente d'autant plus que les concentrations urinaires en arsenic et cadmium s'élèvent.

## Conflits d’intérêts

Les auteurs ne déclarent aucun conflit d'intérêts.
